# Detection of disseminated tumor cells in lymph nodes from patients with early stage non-small cell lung cancer

**DOI:** 10.1186/s13000-016-0504-4

**Published:** 2016-06-17

**Authors:** Ane Kongsgaard, Kjetil Boye, Øystein Fodstad, Siri Juell, Lars H. Jørgensen, Steinar Solberg, Åslaug Helland, Odd Terje Brustugun, Gunhild Mari Mælandsmo

**Affiliations:** 1 Department of Tumor Biology, Institute for Cancer Research, Oslo University Hospital, The Norwegian Radium Hospital, PO Box 4953, Nydalen, Oslo, NO-0424 Norway; 2 Department of Oncology, Oslo University Hospital, The Norwegian Radium Hospital, Oslo, Norway; 3 Department of Cardiovascular and Thoracic Surgery, Oslo University Hospital Rikshospitalet, Oslo, Norway; 4 Department of Genetics, Institute for Cancer Research, Oslo University Hospital, The Norwegian Radium Hospital, Oslo, Norway; 5 Department of Pharmacy, Faculty of Health Sciences, University of Tromsø, Postboks 6050 Langnes, Tromsø, 9037 Norway

**Keywords:** NSCLC, Disseminated tumour cells, Lymph nodes, Immunomagnetic selection, Prognosis

## Abstract

**Background:**

The regional lymph node involvement is a major prognostic factor in patients with non-small cell lung cancer (NSCLC) undergoing surgical resection. Disease relapse is common, suggesting that early disseminated disease is already present in the regional lymph nodes at the time of surgery, and that the current nodal staging classification might be suboptimal. Early detection of disseminated tumor cells (DTCs) in lymph nodes could potentially enable identification of subcategories of patients with high risk of disease relapse.

**Method:**

Lymph node samples were collected from 128 NSCLC patients at the time of surgery and the presence of DTCs determined by immunomagnetic selection (IMS) using the MOC31 antibody recognizing EpCAM. Results obtained with IMS were compared to the pathological staging obtained by histopathology. Associations between the presence of DTCs and clinicopathological variables and patient outcome were investigated.

**Results:**

DTCs were detected in 40 % of the lymph node samples by IMS. Their presence was significantly associated with pN status as assessed by histopathology, and samples from 83 % of the patients with lymph node metastases (pN1-2) had detectable DTCs. In the group of patients who were negative for lymph node metastases by standard histopathology (pN0) DTCs were detected in 32 %. The presence of DTCs was not associated with any other clinicopathological variables. Patients with IMS-positive samples showed decreased relapse free survival compared to patients with IMS-negative samples, but the difference was not statistically significant. The pN status was significantly associated with both relapse free and overall survival, but the presence of DTCs had no prognostic impact in the subcategory of patients with pN0 status.

**Conclusion:**

Our findings do not support further development of lymph node DTC detection for clinical use in early stage NSCLC.

## Background

Curatively intended surgical resection is the standard therapy for operable patients with early-stage non-small cell lung cancer (NSCLC), and the prognosis of these patients is closely related to disease stage [1]. The regional lymph node involvement is a major prognostic factor, and for complete surgical resection of NSCLC a systematic nodal dissection is recommended [2]. This allows pathological staging of the disease according to standardized definitions, and thereby decision of further treatment strategies. The fact that approximately half of the patients undergoing surgery experience disease relapse, suggests that disseminated tumor cells (DTCs) may be present already at the time of surgery [3]. In routine clinical practice, pathological evaluation of resected lymph nodes is done by standard histopathology, a method by which DTCs cannot be identified. The high recurrence rate after surgical resection of NSCLC indicates that current staging classifications are not able to accurately predict patient outcome and that the nodal staging might be suboptimal. Detection of DTCs to regional lymph nodes at the time of surgery could possibly facilitate identification of subcategories of patients with high risk of disease relapse, and thereby stratification of patient groups for adjuvant therapy.

Occult metastatic spread to the lymph nodes or distant sites has been the focus of research over many years, and has been reported under different terminology. The Union for International Cancer Control (UICC) has defined micrometastasis as clusters of tumor cells measuring between 0.2 and 2 mm in diameter, and isolated tumor cells as single tumor cells or small clusters of cells smaller than 0.2 mm [4]. Tumor cells that have spread to lymph nodes or bone marrow are often referred to as DTCs, whereas circulating tumor cells (CTCs) are used for single cells in blood [5]. A number of previous studies have addressed the prognostic value of detecting micrometastasis and DTCs in lymph nodes of NSCLC patients [6–23], but due to considerable differences in terminology, methodology and results, no conclusion can be drawn based on the existing literature. The methods used for detection have traditionally been immunohistochemistry (IHC) with antibodies targeting epithelial-specific proteins like cytokeratins [6–18], and molecular methods using RT-PCR for detection of tumor- or epithelial cell specific mRNA transcripts [3, 19–25]. Our group has previously published a study where we investigated the presence of DTCs in bone marrow aspirates from patients undergoing lung cancer surgery by the use of immunomagnetic selection (IMS) [26]. In the present study we have used IMS to investigate the presence of DTCs in lymph node tissue prospectively collected from patients with early stage NSCLC undergoing curatively intended surgery. In the IMS method small magnetic particles coated with the antibody MOC31 which recognizes the epithelial marker EpCAM, are used to isolate tumor cells from the lymph nodes, allowing fast screening of as much as 2 × 10^7^ cells. The objective of our study was to determine the incidence of lymph node DTCs, and to compare results obtained with IMS to the pathological staging obtained by histopathology. Additionally, we wanted to investigate the associations between the presence of DTCs and clinical and histopathological variables, as well as to patient outcome.

## Methods

### Patients

Between November 2011 and September 2013 we included 183 patients with assumed or verified NSCLC who underwent curatively intended surgical resection at Rikshospitalet, Oslo University Hospital. The project was accepted by the institutional review board and Regional Ethics Committee (S-06402b), the patients received oral and written information and signed a consent form before entering the project. At the time of surgery all patients underwent radical resection of the primary tumor and dissection of the hilar and mediastinal lymph nodes. The excised lymph nodes were first divided into two parts, leaving half the node for routine pathological review and one half for research projects. One node from one lymph node station per patient was made available for IMS analysis. Fifty-five patients were excluded from the study for the following reasons: histology other than NSCLC (13), inadequate material for analysis (41) and withdrawn consent (1). The total study population thus included 128 patients with histologically verified primary NSCLC. The resected tumor tissue and lymph nodes were processed for routine histopathological assessment, and the histological subtypes were classified according to WHO criteria [27]. Tumors were staged according to the Union for International Cancer Control (UICC), TNM 7, and histopathological parameters were retrieved from the pathology reports. After surgery, the patients were followed by clinical evaluation and radiological examination in their corresponding local hospitals according to national guidelines. We collected the follow-up information from the patients’ local hospitals. Survival data were obtained from the National Registry of Norway and updated on January 30th, 2015.

### Immunomagnetic selection

**Fig. 1:**
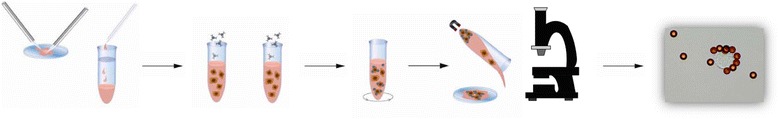
Schematic overview of the IMS technique. Fresh lymph node samples were disintegrated in sterile PBS, followed by filtration, centrifugation, dilution and aliquotation of the cell suspensions. The cells were then mixed with either MOC31 coated magnetic beads (anti-EpCAM) or beads without antibodies for control. The samples were incubated under continuous rotation at 4 °C for 30 min, and placed on a magnet to separate bead-bound and unbound cells. The supernatant containing the unbound cells was removed, and fractions of the cell suspension were examined in the light microscope

The immunomagnetic selection was performed as described previously [28, 29], and a schematic drawing of the method is shown in Fig. 1. The lymph node tissue was placed in a petri dish containing 10 ml phosphate buffered saline (PBS) containing 1 % human serum albumin (HSA) for disaggregation using scalpels. The disaggregated tissue was then filtered through a 70 μm cell strainer (BD Biosciences, Franklin Lakes, NJ, USA) into a 50 ml tube, and the cell strainer was washed with PBS/1 % HSA. The filtered cell suspension was concentrated by centrifugation at 500 g for ten minutes. The cells were then resuspended in 1 ml PBS/1 % HSA, and transferred to round bottom tubes for incubation with antibody-coated beads. Dynabeads M450 sheep anti-mouse (SAM) IgG1 (Dynal, Oslo, Norway) were coated with MOC31 antibody (batch # 80.000, IQ products, Groningen, The Netherlands). Uncoated beads for control experiments were prepared in the same manner, without adding the MOC31 antibody. Magnetic immunobeads with or without the primary antibody were added to tubes containing the cell suspension in a final volume of 1 ml with a bead:cell ratio of 1:2. The samples were then incubated on a rotating mixer for 30 min at 4 °C. After incubation the samples were diluted in PBS/1 % HSA and exposed to a strong magnet for 3 min to separate bound and unbound cells. The supernatant containing the unbound cells was decanted off with the tubes still in the magnet holder, and the positive fraction was resuspended in PBS/1 % HSA. Fractions of 20 μl from the cell suspension were then directly examined in the light microscope to identify and count the number of cells with membrane-bound beads. The cells were also evaluated with respect to size, morphology and three-dimensional shape. Cells were considered EpCAM^+^ if they had five or more immunomagnetic beads attached to the surface, and had diameters exceeding the width of two beads.

### Statistical analysis

Associations between the presence of lymph node DTCs and clinicopathological variables were tested using two-tailed Fisher’s exact test or linear by linear association chi-square test. For analyses of associations between the number of DTCs detected by IMS and clinicopathological variables, two sample *t*-test or one-way ANOVA test, as appropriate, was used. Survival curves were estimated according to the Kaplan-Meier method, and differences between the curves were compared using the log rank test. Survival was measured from date of surgery until date of diagnosis of recurrence or metastasis for relapse free survival, and from date of surgery until death date for overall survival. Cox proportional hazards regression was used to estimate hazard ratios and 95 % confidence intervals (CI). SPSS statistical software version 18.0 (SPSS Inc., Chicago, IL, USA) was used for all the statistical analyses. *P*-values < 0.05 were considered statistically significant.

## Results

### Characteristics of the patient cohort

The clinical and histopathological baseline data of the study cohort are summarized in Table 1. The study cohort consisted of 44 % female and 56 % male patients, with a mean age of 66 years (range 42 – 85). Adenocarcinoma was the most frequent histological subtype (57 % of patients), followed by squamous cell carcinoma (39 %) and large cell carcinoma (4 %). Sixty percent of patients were in pTNM stage I, 27 % of patients were in pTNM II, 10 % in pTNM III and 4 patients (3 %) were in stage IV. As assessed by standard histopathology the majority of patients (80 %) were negative for lymph node metastases and staged as pN0. Among the 26 patients in whom lymph node metastases were detected, 14 % were categorized as pN1 and 6 % as pN2.

**Table 1:** Demographics of patient cohort (*N* = 128)

Parameter		Patients	
		N	%
Sex	Male	71	56
	Female	57	44
Age at surgery	<65 years	52	41
	> 65 years	76	59
Histology	Adenocarcinoma	73	57
	Squamous cell carcinoma	50	39
	Large cell carcinoma	5	4
UICC stage / pTNM7	I	77	60
	II	34	27
	III	13	10
	IV	4	3
pT	pT1	41	32
	pT2	61	48
	pT3	25	19
	pT4	1	1
pN	pN0	102	80
	pN1	18	14
	pN2	8	6
Tumour size (cm)	≤ 2.0 cm	32	25
	2.1–3.0 cm	27	21
	3.1–5.0 cm	46	36
	5.1–7.0 cm	10	8
	>7.0 cm	13	10
Tobacco use	Current smoker	77	60
	Former smoker	46	36
	Never smoker	6	5

### Detection of DTCs by IMS

In nine cases the sampled lymph node station was not registered, resulting in a total of 119 cases for the methodological analyses. Based on our experience with IMS and our previous publication [26], the cutoff for a positive sample was 10 cells, meaning that a sample with 11 or more EpCAM^+^ cells was classified as IMS-positive. In 47 of 119 samples (40 %) > 10 EpCAM^+^ cells were detected, and the median number of cells in the IMS-positive samples was 61 (range 14 – 200). Between 11 and 50 EpCAM^+^ cells were detected in 45 % of positive samples, 51 – 100 EpCAM^+^ cells were detected in 28 % and ≥ 100 cells were detected in 28 % of the positive samples. Associations between the presence of EpCAM^+^ cells and the clinicopathological characteristics of the patients are summarized in Table 2. The presence of EpCAM^+^ cells was significantly associated with pN status (*p* < 0.001, Table 2). Lymph node samples from 9/11 (82 %) pN1 patients and 5/6 (83 %) pN2 patients, respectively, were IMS-positive. Three of 17 patients (18 %) in whom lymph node metastases were detected by histopathology (pN1-2) were categorized as IMS-negative. In the subcategory of patients who were negative for lymph node metastases by histopathology (pN0), 32 % (33/102) were IMS-positive (Table 2). The exact number of EpCAM^+^ cells in each sample was also significantly associated with pN status (*p* < 0.001, one-way ANOVA test). In fact, 4/6 (67 %) of patients with pN2 status had more than 100 EpCAM^+^ cells in their samples, compared to only 8/102 (8 %) of the patients with pN0 status. Analyzing the relationship between the IMS results and the other clinicopathological parameters revealed no association with gender, age, histology, pTNM, pT status, tumor size or tobacco smoking habits (Table 2).

**Table 2:** Relationship between the presence of DTCs as detected by IMS and clinicopathological variables (*N* = 119 ^a^)

Parameter		N^b^	%^b^	*p*-value
Gender	male	28	44	
	female	19	35	0.36
Age at surgery	< 65 years	22	46	
	> 65 years	25	35	0.26
Histology	ADC	26	38	
	SCC	19	40	
	LCC	2	50	0.89
pTNM	I	26	34	
	II	13	45	
	III	7	78	
	IV	1	25	0.12
pT	pT1	20	51	
	pT2	19	33	
	pT3	8	38	
	pT4	0	0	0.15
pN	pN 0	33	32	
	pN I	9	82	
	pN II	5	83	< 0.001
Tumor size (cm)	≤ 3.0 cm	26	47	
	≥ 3.1 cm	21	33	0.13
Tobacco	Never smoker	2	33	
	Former smoker	20	47	
	Current smoker	25	36	0.54

^a^Nine patients excluded from analysis due to uncertain lymph node station

^b^The number and percentage of positive samples are shown

### Patient outcome and associations with clinicopathological parameters

For the follow-up and survival analyses the four patients with stage IV disease were excluded, resulting in a cohort of 124 patients. Median follow-up time for patients still alive who had not developed metastasis or local recurrence was 26 months (range 16 –38). Twenty-five of 124 patients (20 %) developed disease relapse, while 24 patients (19 %) died during the follow-up period. Associations between clinicopathological variables and patient outcome are shown in Table 3. Univariate analyses revealed that pTNM stage and pN status were significantly associated with disease relapse (*p* < 0.001 and *p* = 0.001, respectively) and with overall survival (*p* = 0.002 and 0.005, respectively). There was a significant association between patient outcome and gender, with a 2-year relapse free survival rate of 87 % for females, compared to 70 % for males (*p* = 0.03). A relationship between histological subtype and patient outcome was also detected, however this result was caused by the fact that three out of four patients with large cell carcinoma relapsed, and should be interpreted with caution due to the small number of patients in the subcategory.

**Table 3:** Univariate analysis of associations between clinicopathological variables and patient outcome (*N* = 124^a^)

	Relapse free survival			Overall survival		
Parameter	*p*	Hazard ratio	(95 % CI)	*p*	Hazard ratio	(95 % CI)
Gender	0.03			0.9		
Female						
Male		2.7	(1.1–6.8)		1.1	(0.5–2.4)
Age	0.06			0.3		
> 65 years						
< 65 years		2.8	(0.9–8.6)		1.5	(0.7–3.3)
Histology	0.01			0.04		
Adenocarcinoma						
Squamous cell carcinoma		0.7	(0.3–1.8)		0.5	(0.2–1.2)
Large cell carcinoma		9.6	(2.7–34.0)		4.7	(1.1–20.1)
pTNM	< 0.001			0.002		
I						
II		1.7	(0.6–4.5)		2.2	(0.8–5.7)
III		8.8	(3.4–22.4)		7.1	(2.6–19.2)
pT	0.07			0.02		
pT1					1.7	(0.6–5.6)
pT2		2.4	(0.8–7.2)		5.6	(1.7–18.2)
pT3		4.8	(1.4–16.3)			
pT4						
pN	0.001			0.005		
pN0						
pN1		3.6	(1.4–9.1)		3.5	(1.4–8.9)
pN2		8.2	(2.9–23.0)		5.1	(1.7–15.8)
Tumor size	0.18			0.73		
≤ 3.0 cm						
≥ 3.1 cm		1.7	(0.8–3.9)		1.2	(0.5–2.6)
DTCs detected by IMS	0.18			0.77		
IMS negative						
IMS positive		1.7	(0.8–3.8)		1.1	(0.5–2.5)

^a^Four patients were excluded from survival analyses due to stage IV disease

### Associations between DTCs and patient outcome

**Fig. 2:**
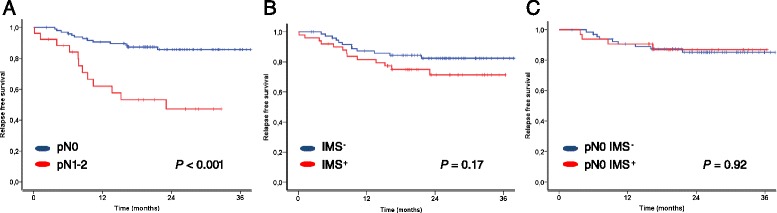
Kaplan-Meier survival plots depicting relapse-free survival based on pathological lymph node status (**a**), and based on the presence of DTCs as detected by IMS in the overall patient cohort (**b**) and in the pN0 patient cohort (**c**)

There was a small, non-significant difference in relapse free survival between patients with IMS-negative and IMS-positive lymph node samples (*p* = 0.17, Fig. 2b and Table 3). Two-year relapse free survival for IMS-negative patients was 82 %, compared to 71 % for IMS-positive patients. There was no association to overall survival (*p* = 0.77, Table 3). Twenty-six percent (13/50) of patients with IMS-positive samples developed disease relapse during the follow-up period, compared to 16 % (12/74) of patients with IMS-negative samples. The exact number of EpCAM^+^ cells detected by IMS was not associated with disease relapse (*p* = 0.57, Cox univariate analysis). We performed subcategory analyses including only patients who were classified as pN0 by histopathology, but found no difference in relapse free survival (*p* = 0.92, Fig. 2c) or overall survival (*p* = 0.39, data not shown) between IMS-negative and IMS-positive patients. Similar to the whole patient cohort, the exact number of EpCAM^+^ cells was not related to disease relapse in this subcategory (*p* = 0.57, Cox univariate analysis). Finally, dividing patients into subcategories according to the number of EpCAM^+^ cells in the sample did not reveal any associations with outcome.

## Discussion

In the present study we have examined the presence and prognostic implication of DTCs in prospectively collected lymph node samples from early stage NSCLC patients undergoing surgical resection. By using the technique IMS with the EpCAM-targeting antibody MOC31, we found a strong association between pN status as assessed by standard histopathology and detection of EpCAM^+^ cells. The pN status, but not the IMS result, was significantly associated with patient outcome. Among the patients staged as pN0 by histopathology, 32 % were categorized as IMS-positive, but IMS was not able to predict prognosis in this patient subcategory. Taken together, we conclude that detection of lymph node DTCs by IMS does not improve nodal staging in NSCLC patients, and cannot be used for identification of patients with high risk of relapse after surgical resection.

A number of publications have investigated the incidence of micrometastatic disease or DTCs in lymph nodes from patients with NSCLC, the majority using IHC or RT-PCR, with conflicting results. Several groups have shown an association between the presence of micrometastasis or DTCs and poor prognosis [7, 8, 10, 11, 13, 14, 19–23] while other have concluded like in our study, that their presence has no impact on patient outcome [15–18]. Data from the diverse publications are not easily comparable due to differences in methodology as well as in size and composition of patient cohorts. Also, various definitions of the terms “micrometastases”, “occult tumor cells” and “isolated tumor cells” have been used, and many authors who demonstrated negative prognostic value did not specify whether they investigated isolated tumor cells or true micrometastasis [6, 14, 30]. In 2010, Herpel et al. [8] showed that nodal micrometastasis specifically defined as small tumor deposits had prognostic significance, however two other studies investigating isolated tumor cells in regional lymph nodes concluded that this did not affect survival in resected NSCLC patients [16, 17]. One might hypothesize that NSCLC patients with detectable single DTCs have similar prognosis to patients with node negative disease, while patients with micrometastasis may have higher risk of relapse. Interestingly, similar results have been found in breast cancer [31]. In a paper from 2008, Marchevsky et al. investigated both isolated tumor cells and micrometastases in lymph nodes from 266 stage I NSCLC patients, and concluded that the presence of neither of these were significantly associated with survival. The authors also performed a meta-analysis including their own data and 13 other studies, and concluded on no significant association between micrometastasis and survival [18]. Thus, based on the current literature and our own results, detecting DTCs in regional lymph nodes does not seem to provide a clinically useful method for identifying subcategories of NSCLC patients with high risk of disease relapse. Previously we have also shown that detection of DTCs in bone marrow by IMS has no prognostic impact in early stage NSCLC patients [26].

The present study is, to our knowledge, the first to use IMS as method for detection of DTCs in regional lymph nodes from NSCLC patients. This technique was developed in our lab, and has been used in several published papers investigating DTCs in bone marrow [26, 28, 32–35] and lymph nodes [36] in various cancer types. Evaluation of morphology, size and shape of the MOC31-bead-bound cells is challenging when using IMS, and is dependent on the observer’s skills and experience. In our study we found a high number of MOC31-bead bound cells in the samples, and the cut off value for an IMS-positive sample was set at 10 cells based on experience and our previous publication [26]. The high number of discovered cells might indicate that some nonmalignant cells are also detected with this method, and in fact lymph node reticulum cells can express epithelial antigens [37, 38], and weak binding of MOC31 to lymphocytes has been described [39]. Interestingly, however, in a study using RT-PCR to identify tumors cells in lymph nodes in a NSCLC cohort comparable to ours, Nordgård et al. also reported a high number of positive findings in histopathologically node negative patients [40]. Another important issue when using antibodies targeting EpCAM is that the expression of this protein may vary in tumor cells during disease progression [41], and expression can be down-regulated as a consequence of epithelial-to-mesenchymal transition (EMT) [42]. This might perhaps explain why no EpCAM^+^ cells were discovered in 3 of 17 lymph node samples from patients who had metastases detected by routine histopathology. There are clearly several methodological issues associated with the use of IMS, leading to considerable challenges for the further development and application of this method.

Our study is limited by the fact that we only had access to one lymph node sample from one node station per patient. Analyzing a number of nodes from various stations from each patient would of course have been preferable. Moreover, a larger patient cohort and longer follow-up period would increase the reliability of our results. However, we were able to detect EpCAM^+^ cells in the vast majority of samples from lymph nodes in which metastases had been shown on histopathology, and there was a strong association between results from the IMS method and nodal staging by standard histopathology.

## Conclusion

We conclude that detecting EpCAM^+^ cell in regional lymph nodes by IMS does not predict the prognosis of resected NSCLC patients, as the presence of such cells in patients who were lymph node negative showed no association with reduced survival. Our findings do not support further development of assays for detection of DTCs in lymph nodes for clinical use in NSCLC.

## Abbreviations

NSCLC, non-small cell lung cancer; DTCs, disseminated tumor cells; IMS, immunomagnetic selection; UICC, Union for International Cancer Control; PBS, phosphate buffered saline; HSA, human serum albumin; CTCs, circulating tumor cells; IHC, immunohistochemistry
